# Osteopontin induces growth of metastatic tumors in a preclinical model of non-small lung cancer

**DOI:** 10.1186/1756-9966-31-26

**Published:** 2012-03-23

**Authors:** Farbod Shojaei, Nathan Scott, Xiaolin Kang, Patrick B Lappin, Amanda A Fitzgerald, Shannon Karlicek, Brett H Simmons, Aidong Wu, Joseph H Lee, Simon Bergqvist, Eugenia Kraynov

**Affiliations:** 1 Pfizer Global Research and Development, Department of Oncology, La Jolla, CA, USA; 2 Pfizer Global Research and Development, Department of Pharmacokinetics, Dynamics, and Metabolism, La Jolla, CA, USA; 3 Pfizer Global Research and Development, Department of Drug Safety Research & Development, La Jolla, CA, USA; 4 Pfizer Global Research and Development, Department of Global Biotherapeutics Technologies, Cambridge, MA, USA

## Abstract

Osteopontin (OPN), also known as SPP1 (secreted phosphoprotein), is an integrin binding glyco-phosphoprotein produced by a variety of tissues. In cancer patients expression of OPN has been associated with poor prognosis in several tumor types including breast, lung, and colorectal cancers. Despite wide expression in tumor cells and stroma, there is limited evidence supporting role of OPN in tumor progression and metastasis. Using phage display technology we identified a high affinity anti-OPN monoclonal antibody (hereafter AOM1). The binding site for AOM1 was identified as SVVYGLRSKS sequence which is immediately adjacent to the RGD motif and also spans the thrombin cleavage site of the human OPN. AOM1 efficiently inhibited OPNa binding to recombinant integrin αvβ3 with an IC50 of 65 nM. Due to its unique binding site, AOM1 is capable of inhibiting OPN cleavage by thrombin which has been shown to produce an OPN fragment that is biologically more active than the full length OPN. Screening of human cell lines identified tumor cells with increased expression of OPN receptors (αvβ3 and CD44v6) such as mesothelioma, hepatocellular carcinoma, breast, and non-small cell lung adenocarcinoma (NSCLC). CD44v6 and αvβ3 were also found to be highly enriched in the monocyte, but not lymphocyte, subset of human peripheral blood mononuclear cells (hPBMCs). *In vitro*, OPNa induced migration of both tumor and hPBMCs in a transwell migration assay. AOM1 significantly blocked cell migration further validating its specificity for the ligand. OPN was found to be enriched in mouse plasma in a number of pre-clinical tumor model of non-small cell lung cancers. To assess the role of OPN in tumor growth and metastasis and to evaluate a potential therapeutic indication for AOM1, we employed a Kras^G12D-LSL^p53^fl/fl ^subcutaneously implanted *in vivo *model of NSCLC which possesses a high capacity to metastasize into the lung. Our data indicated that treatment of tumor bearing mice with AOM1 as a single agent or in combination with Carboplatin significantly inhibited growth of large metastatic tumors in the lung further supporting a role for OPN in tumor metastasis and progression.

## Introduction

OPN is a multifunctional protein involved in several pathological processes such as inflammation and cancer [1]. As an acidic glycophosphoprotein, OPN contains a RGD (arginine-glycine-aspartate) integrin binding motif, a hydrophobic leader sequence (indicative of its secretory characteristic), a thrombin cleavage site adjacent to RGD domain, and a cell attachment sequence [2]. OPN has been found to be present in three forms in tissues and fluids: i) an intracellular protein in complex with hyaluronan-CD44-ERM (ezrin/radixin/moesin) that is involved in migration of tumor and stromal cells [3]; ii) an extracellular protein that is abundant at mineralized tissues [4]; iii) a secreted protein that is found in fluids isolated from metastatic tumors [5] and also found in organs such as placenta [6,7], breast [8], and testes [9]. At the protein synthesis level, OPN undergoes extensive post-translational modification including phosphorylation and glycosylation [10]. Additionally, there are three splice variants of OPN (OPNa, OPNb, and OPNc) that may have distinct characteristics in different tissues and tumor types [11]. For example, OPN-c has been suggested to be expressed in invasive breast tumors and is highly correlated with patient's survival in HER-2 breast patients [12]. Irrespective of OPN isoform, a series of other studies have suggested a role for plasma OPN as a biomarker of tumor progression in colon [13,14], lung [15], and prostate cancers [16,17].

The RGD sequence in OPN protein enables it to bind to CD44-ERM and several integrins including α_V_β_1_, αvβ3, and α_V_β_5 _[18]. Given the wide expression of integrins and CD44, both cancer cells as well as stromal compartment are targeted by OPN in the tumor mass. Binding of OPN to the above receptors on tumor cells triggers downstream signaling pathways including Ras, Akt, MAPK, Src, FAK and NF-KB [1] that collectively lead to the following in tumor cells: i) invasion to ECM (extracellular matrix) mainly via upregulation of MMPs [19] (matrix metalloproteinases) and uPAs [20] (urokinase plasminogen activator) by OPN; ii) increased migration and adhesion of tumor cells [21]; iii) inhibition of cell death likely through upregulation of anti-apoptosis mediators such as GAS6 [22]; and iv) development of pre-metastatic niche [23]. Additionally, tumor stroma such as endothelial cells [18] and immune infiltrating cells [24,25] (particularly monocytes) express OPN receptors. Angiogenesis is proven to be a critical component of tumor mass by supplying oxygen and nutrients for cancer cells [26]. Angiogenesis in the tumor is induced by OPN directly by binding to αvβ3, and/or indirectly via upregulation of VEGF (vascular endothelial growth factor) [27,28]. Additionally, OPN may suppress immune response via inhibition of iNOS (inducible nitric oxide synthase) in immune infiltrating cells further creating a conducive microenvironment for growth and invasion of tumor cells [29,30]. It is noteworthy to mention that cleavage by thrombin enhances biological activity of OPN [31] through increased exposure of N-terminal domain to integrin binding sites [32] and/or via formation of a complex between the c-terminal domain and cyclophilline and CD147 resulting in the activation of Akt1-2 and MMP-2 [33]. VEGF may accelerate thrombin activity to generate cleaved-OPN that in turn results in increased migration of endothelial cells [34].

To further understand the role of OPN in tumor progression, we screened phage display libraries and identified a monoclonal anti-OPN antibody (AOM1) capable of neutralizing human and mouse OPN. In vitro, AOM1 inhibited OPN-induced migration of tumor cells and monocytes. Furthermore, AOM1, as a single agent or in combination with a cytotoxic agent, inhibited growth of large tumors in the lung in a metastatic model of NSCLC indicating a role for OPN in lung metastasis.

## Materials and methods

### Inhibition of thrombin mediated degradation of human OPN

Ability of AOM1 to inhibit OPN cleavage by thrombin was evaluated in a western blot assay. Reaction buffer included PBS pH 7.2 containing 2 mM MgCl_2 _and 0.2 mM MnCl_2_. Both AOM1 and the control antibodies were added to human OPN (2.2 μg/ml) and reaction buffer to a total volume of 900 μl. Anti-OPN antibody concentration was titrated from 3 nM to 1000 nM. OPN and AOM1 were pre-incubated at 37°C on a rotary shaker for 1 hour to allow association to occur. Next, 100 ul of 50% thrombin-agarose slurry (in reaction buffer, Sigma, CA) was added to the reaction mixture and were incubated for 2 hours at 37°C on a rotary shaker. Reaction mixture supernatant was removed and analyzed by SDS-PAGE and western blot using a mouse anti-human OPN antibody (34E3, IBL, Japan) specific to the N-terminal fragment of thrombin cleaved OPN. Intensity of the western blot staining of the thrombin cleaved N-terminal fragment was compared at different concentrations of AOM1 to approximate an IC50 for thrombin cleavage inhibition.

### Integrin binding inhibition assay

Immunosorbent plates (COSTAR Corning, CA) were coated with 100 μl/well integrin αVβ3 (10 μg/ml, R&D System, MN) in Buffer 1 (PBS 7.2 with 0.2 mM MnCl2 and 2 mM MgCl2) for overnight at 4°C. Plates were then washed three times with Buffer 1 and non-specific binding sites blocked with 200 μl/well of blocking buffer (3% BSA in Buffer 1) for two hours at 37°C. Next, plates were washed three times with Buffer 1 and 100 μl of OPN/test antibody mixture was applied to the plate surface. The OPN/test antibody mixture was prepared as follows. Human OPN (R&D systems, MN) was maintained at a constant final concentration (6 μg/ml) in the blocking buffer. OPN was mixed with either AOM1 or control antibody. Antibody concentrations were titrated from 10 μM in a three-fold dilution series to approximately 0.1 nM. Human OPN and test antibody were pre-incubated for 1 hour at room temperature on a rotary mixer before being applied to the αVβ3 coated ELISA plates. After a washing step (3 times with Buffer 1 + 0.05% Tween-20 and three times with Buffer 1 alone), rabbit polyclonal anti-human OPN antibody (O-17, IBL, Japan) was added to the plates (100 μl/well) at a concentration of 4 μg/ml for 1 hour at room temperature. Plates were then washed (3 times with Buffer 1 + 0.05% Tween-20 and 3 times with Buffer 1 alone) and goat-anti-rabbit antibody (Fc specific) HRP conjugate (Jackson Immunoresearch, PA) was added to each well (100 μl/well, 1 in 5000 dilution in Block Buffer) for 1 hour at room temperature. Following final washes (3 times with Buffer 1 + 0.05% Tween-20 and 3 times with Buffer 1 alone) ELISA was developed with 100 μl/well BM Blue POD substrate (Roche, NJ) and the colorimetric reaction was stopped with 100 ul/well 0.2 M H2SO4. Absorbance at 450 nm was measured using a Spectromax plate reader (Molecular Devices, CA) and analysis was conducted using Microsoft Excel Data-Analysis Add-In fitting IC50 curves to a 4-paramter sigmoidal saturation binding model.

### Selectivity of AOM1 for OPN

EIA/RIA plates (Corning, NY) were coated with 1 mg/ml of RGD-motif containing protein which included OPN, Thrombospondin, Vitronectin, ColIAI or Fibronectin (R&D Systems, MN) in Buffer 1 (PBS pH 7.2 containing 2 mM MgClR_2R _and 0.2 mM MnClR_2R _for 16 hours at 4°C). Plates were washed three times with Buffer 1 and were blocked with commercially available Blocking buffer (3% BSA (Rockland, PA) in Buffer 1) followed by washing three times with Buffer 1 and AOM1 was added at 0, 0.1, 1, 10, and 1000 nM in blocking buffer, and incubated at RT for 1 hr. Plates were washed (3 times with Buffer 1 + 0.05% Tween-20 and three times with Buffer 1 alone). Goat Anti-Human IgG (Fc) Peroxidase Conjugate (Jackson Immunoresearch, PA) was added (1 in 5000 in block buffer) and plates were incubated at RT for 1 h followed by a wash (3 times with Buffer 1 + 0.05% Tween-20 and three times with Buffer 1 alone). BM Blue Solution (Roche, NJ) was used to develop the assay and quenched with 0.18 M HR_2R_SOR_4R_. Absorbance at 450 nm was detected using a Spectramax plate reader (Molecular Devices, CA) and data were analyzed using Microsoft Excel.

### Characterization of AOM1 Fab binding to OPN

Binding of Fab fragment of AOM1 to recombinant OPN was determined using surface plasmon resonance (SPR) analysis on a Biacore 3000 instrument (GE Healthcare, CA). Recombinant OPNs (human: 1433-OP-050/CF; mouse 441-OP-050/CF; R&D System, MN) was immobilized on a CM5 biosensor chip using standard EDC/NHS amine coupling chemistry, at 25°C using a 1 μM in 10 mM sodium acetate pH 5.0. Experiments were carried out in a buffer containing 10 mM HEPES pH 7.4, 150 mM NaCl, 0.005% P20 at 25°C using a two-fold dilution series of the Fab. Data were analyzed using the Scrubber2 software (BioLogic Software, Pty., Australia). Injections were referenced to a blank surface and by a buffer blank. Kinetic characteristics were obtained from a fit to a simple kinetic binding model using the Scrubber2 program software (BioLogic Software, Pty., Australia).

### Epitope mapping

Epitope mapping studies were carried out using an overlapping series of synthetic peptides (CPC Scientific, CA) designed based on the primary sequence of OPN. Peptides corresponding to the region 143-172 of human OPN are listed below:

1. 143EVFTPVVPTVDTYDGRGDSVVYGLRSKSKK172

2. 143EVFTPVVPTVDTYDGRGDSVVYGLR167

3. 143EVFTPVVPTVDTYD156

4. 156DGRGDSVVYGLRSKSKK172

Binding of each peptide was determined to the immobilized anti-OPN antibody by SPR. The antibody was immobilized on a CM5 chip by standard EDC/NHS amine coupling chemistry, at 25°C using a 1 μM in 10 mM sodium acetate pH 5.0. Peptides were diluted to 5 uM in 10 mM HEPES pH 7.4, 150 mM NaCl, 0.005% P20 and diluted with a two-fold series. The samples were analyzed at a flow rate of 20 uL/min and were injected serially over all four flow cells for a 5 minute association and a 5 minute dissociation. The binding data were fit to a simple equilibrium binding model using Scrubber2 (BioLogic Software, Pty., Australia).

Migration assay was performed in transwell plates (VWR, CA) using standard protocol provided by the manufacturer. All the cell lines (JHH4, MSTO-211H and MDA-MB435) were purchased from ATCC (American Type Culture Collection; VA) and were grown in RPMI (GIBCO BRL, CA) supplemented with 10% FBS (Sigma Aldrich, CA). Cells were harvested from flasks and were placed (5 × 10^4 Cells in 100 ul plain media) on the top chamber of transwells. Plates were incubated in a cellular incubator for 4 hrs and migrating cells were counted in the bottom well.

To measure migrating hPBMCs, blood samples were taken from healthy individuals under guidelines provided by Pfizer Department of Environmental Health and Safety. Nearly 40 ml blood was collected from a healthy individual in a 4 CPT tube and was span 20 min at 3000 RPM followed by harvesting PBMCs in 50 ml polypropylene tubes, washing twice in plain RPMI1640 and starvation for 2 hrs at 37°C. Cells were then spiked with AOM1 or control antibody and were incubated at 37°C for 1 hr in a cell incubator. Next, 150 ul of pretreated PBMC in RPMI was added to the top chamber of transwell while bottom wells contained either plain RPMI with or without OPN (R&D System, MN, 5 ug/ml). Plates were incubated in a cell incubator for 4 hrs at 37°C and migratory cells were counted in the bottom well.

### Flowcytometry, histology and ELISA

Cells (tumor cells or PBMCs) were stained with anti-CD44v6-APC (R&D System, MN) or anti-αvβ3-PE (R&D System, MN) antibodies using standard protocol provided by the manufacturer. Mouse antibodies (CD44-FITC α_v_-APC and β_3_-PE) were all purchased from BD Biosciences. All the stained samples were analyzed in a Calibur instrument (BD Biosciences, CA) and data were analyzed in FCS express software (De Novo, CA). Whole lungs were collected from treated animals and were preserved in formalin and embedded in paraffin. Sections of lungs were stained with Hematoxylene and Eosin staining (H&E) to evaluate efficacy of different treatments on the growth of lung tumors. Plasma samples were collected when mice were euthanized at the end of *in vivo *study and mouse OPN was measured by an ELISA kit (R&D System, MN) using a protocol provided by the manufacturer.

### Tumor implantation

Kras^G12D-LSL^p53^fl/fl ^mice (n = 10) were inhaled intranasally with Adeno-CMV-Cre (2.5 × 10^7 viral particles, University of Iowa, IO). Using trocar catheter, pieces of tumors were removed from the lungs at 16 weeks post-inhalation and were immediately implanted subcutaneously in Scid/beige mice. Tumor bearing mice (n = 10) were randomized at 8 days post-implantation when tumors reached 200 mm^3 ^using caliper measurement [35]. Randomized animals were treated with vehicle, Carboplatin (25 mg/kg weekly, Hospira, IL), AOM1 (30 mg/kg weekly) and combination of both compounds using intra-peritoneal route of administration. The entire study was terminated when vehicle-treated tumors reached ~2500 mm^3^. Whole lungs were fixed in formalin, embedded in paraffin and were cut using a microtome machine in the laboratory. Slides from each treatment were stained in H&E (hetoxylin and eosin) and metastasis in each section was assessed by a certified pathologist. Lung lesions were quantified based on size of tumors to small (less than 10 cells) medium (10-200) and large (more than 200 cells).

## Results

### Development and characterization of AOM1 monoclonal antibody targeting mouse and human OPN

**Figure 1:**
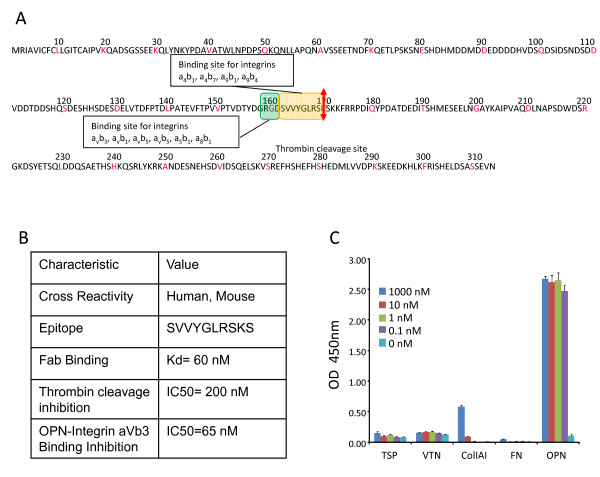
**Development of anti-OPN antibody**. **A **Amino acid sequence of OPNa (full length OPN). Truncated isoforms OPNb and OPNc are highlighted with blue and yellow, respectively. Binding sites for integrins are highlighted with green (RGD binding integrins) and orange (LDV binding integrins). Thrombin cleavage site is marked by a red arrow. **B **Characterization of AOM1 including its cross-reactivity, binding epitope, dissociation constant (K_D_) for the Fab and its ability to inhibit binding of recombinant OPNa to immobilized integrin αvβ3 have been determined. **C **Selectivity of AOM1 for human OPN over other RGD-motif containing proteins was assessed by ELISA as detailed in Materials and Methods. RGD containing proteins were immobilized on an immunosorbent plate and binding of AOM1 assessed at 0.1, 1, 10 and 1000 nM concentrations. With the exception of 1000 nM AOM1 vs. ColA1, there was no binding observed at any concentration of AOM1 up to 1000 nM versus thrombospondin, vitronectin, ColA1 and fibronectin whilst saturated binding was observed *vs*. OPN at antibody concentrations as low as 0.1 nM AOM1. Each bar represents mean OD450 nm value of triplicate measurements with standard error bars.

Analysis of aa (amino-acid) sequences of three different isoforms of OPN (a, b and c) provided some clue about common regions between the isotypes in order to identify antibodies potentially capable of binding and neutralizing all forms of OPN (Figure 1A). Consistent with a published report [36], there is a conserved aa sequence in all three isoforms corresponding to binding sites for a series of integrins including α_4_β_1_, α_4_β_7_, α_9_β_1_, α_9_β_4_, αvβ3, α_v_β_1_, α_v_β_5_, α_v_β_5_, α_5_β_1 _and α_8_β_1 _making it an attractive epitope to target with an anti-OPN neutralizing antibody. Screening of phage display libraries identified several antibodies with the potential to bind to the integrin biding sequence of OPN. Further detailed biochemical and cellular characterization led to the discovery of AOM1, a fully human monoclonal antibody with the ability of neutralizing both human and mouse OPN. Species specificity of AOM1 was determined by SPR (surface plasmon resonance) using OPN immobilized on a Biacore chip. AOM1 was found to cross-react with human and mouse OPN (Figure 1.B). Using a Fab fragment of AOM1, affinity of AOM1 to human OPNa was measured to be 50 nM. Epitope recognized by AOM1 on human OPN was determined using a series of overlapping synthetic peptides corresponding to the region 143-172 of human OPN. AOM1 binds to SVVYGLRSKS motif which is a binding site for integrins α_4_β_1_, α_4_β_7_, α_9_β_1_, and α_9_β_4R _(Figure 1). The epitope is immediately adjacent to the RGD sequence which is the binding site for another family of integrins (αvβ3, α_v_β_1_, α_v_β_5_, α_v_β_5_, α_5_β_1 _and α_8_β_1_). In addition, the AOM1 binding epitope spans over the main thrombin cleavage site on OPN. The ability of AOM1 to inhibit OPN binding to integrin αvβ3 which is considered to be the major receptor by which OPN regulates cancer cell migration and proliferation, and to prevent thrombin-mediated cleavage of OPN was characterized in an ELISA-based and western blot assays, respectively. In both cases AOM1 demonstrated high inhibitory activity (Figure 1B&C). Therefore, this unique binding epitope allows AOM1 to inhibit multiple functional activities of OPN by preventing signaling through integrins as well as blocking cleavage of OPN by thrombin which has been shown to produce functionally more active OPN fragments than the full length molecule. Of note, AOM1 has high selectivity for OPN and does not recognize other RGD containing proteins which is consistent with its binding epitope.

### OPN acts as a chemotactic agent for human tumor cells and monocytes

**Figure 2:**
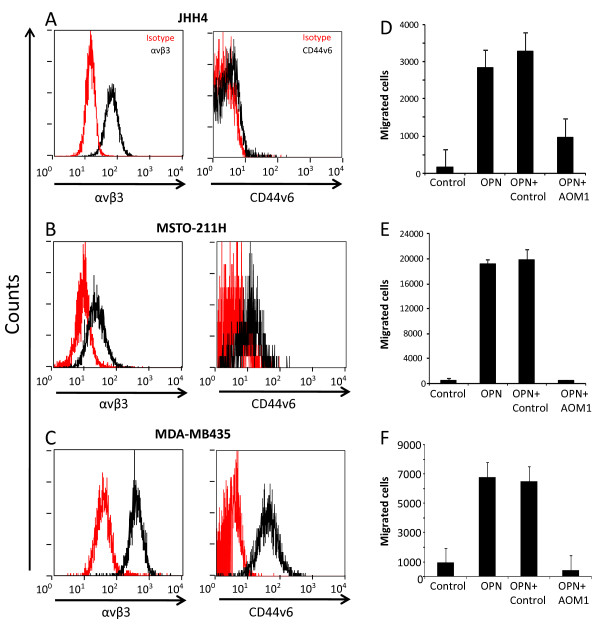
**OPN act as a chemotactic factor in human cells lines expressing OPN receptors**. **A-C **Using flowcytometry expression of OPN receptor, mainly CD44v6 and αvβ3 was assessed in series of human cell lines. Three cell types found to have greater expression of one or both receptors. These lines include JHH4 hepatocellular (A) carcinoma, MSTO211H mesothelioma (B) and MDA-MB435 melanoma cells (C). **D-F **Migration assay provided functional relevance for expression of OPN receptors in the above cell lines. Using transwell, each cell line was added to the top chamber and its migration towards OPN was evaluated.

To identify a potential therapeutic indication for AOM1 we first screened a series of human and mouse cancer cells to identify cell lines that express OPN receptors in particular αvβ3 and CD44v6. As illustrated in Figure 2A-C, FACS analysis identified at least three cell lines expressing OPN receptors including JHH4, MDA-MB435, and MSTO-211H. Furthermore, transwell assay data showed that these cells were capable of migrating to OPN (5 μg/ml) indicating a functional relevance for receptor expression in these cells (Figure 2D-F). Treatment with AOM1 (150 μg/ml) fully inhibited cell migration suggesting that blockade of integrin binding site is sufficient to inhibit cell migration to OPN.

**Figure 3:**
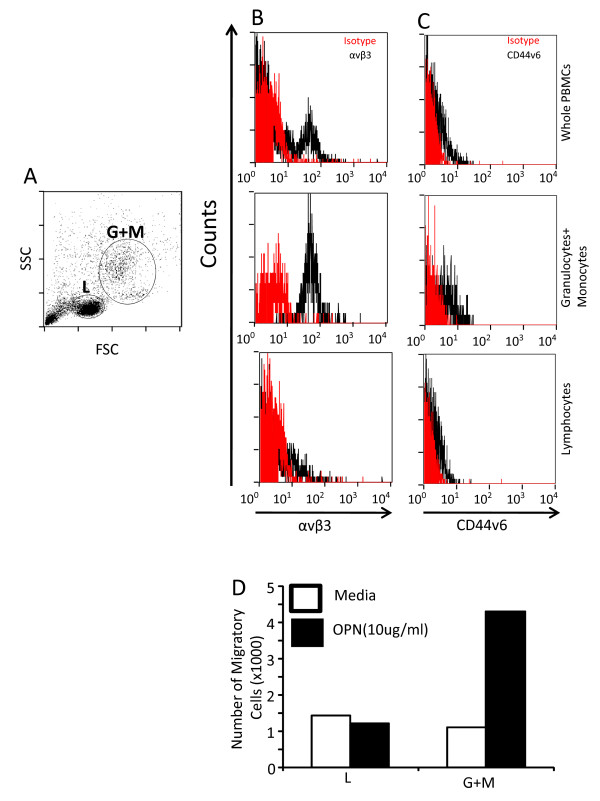
**CD44v6 and αvβ3 are highly expressed in granulocyte and monocyte but not lymphocyte subpopulation of hPBMCs**. **A **Representative side scatter vs. forward scatter plot of hPBMCs representing populations of lymphocytes (L), granulocytes (G) and monocytes (M). **B&C **Expression of OPN receptors (αvβ3 (B) and CD44v6 (D)) was measured in hPBMCs and was evaluated in L vs. GM subsets. **D **Transwell migration assay in L vs. GM subset indicated that only the latter is capable of migrating toward OPN thus providing a functional relevance of expression of receptors.

In addition to tumor cells, we investigated expression of OPN receptors in human PBMCs (peripheral blood mononuclear cells; Figure 3A). Flowcytometry data indicated expression of αvβ3 and to a lesser extent CD44v6 in the entire human PBMCs (Figure 3B). Further gating on populations of granulocytes and monocytes (GM) vs. lymphocytes showed a greater expression of both receptors in GM compared to lymphocyte subset (Figure 3C). The migration assay supported flowcytometry data since only GM, but not lymphocytes, migrated towards OPN (Figure 3D). Overall, and consistent with published reports [37], we have provided receptor expression and functional data further supporting a role for OPN in tumor growth via affecting both cancer cells and stroma.

### OPN is highly enriched in a murine model of NSCLC

In addition to human cells we also analyzed mouse cell lines to identify a preclinical model to test efficacy of AOM1 with specific focus on lung tumors. OPN has been shown to be highly enriched in lung tumors [38]. Surgical removal of primary lung tumors in patients results in a significant reduction in levels of OPN in plasma further indicating a role for OPN as a biomarker of tumor progression in NSCLC [39]. Consistent with these findings, a mass spectrometry method was developed to quantify three different isoforms of OPN (a, b, and c) in plasma samples obtained from NSCLC patients and healthy individuals. Analysis of plasma samples showed that all three isoforms of OPN were present in healthy individuals but were less abundant than in cancer patients. Of note, elevated OPNa accounted for the majority of the increased total OPN in cancer patients [40].

**Figure 4:**
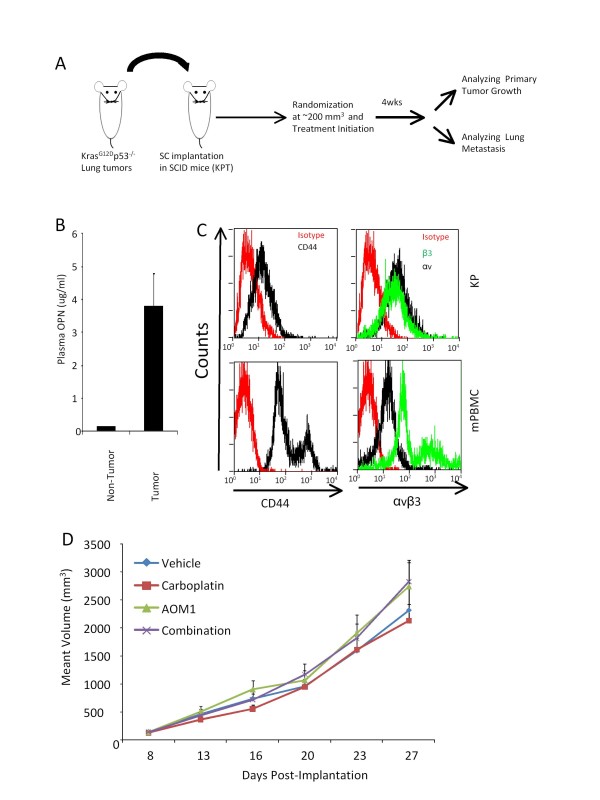
**Characterizing OPN and its receptors in mouse NSCLCs**. **A **Development of KPT model. Kras^G12D-LSL^p53^fl/fl ^(KP) mice were inhaled with Adeno-CMV-Cre at approximately 8 weeks after birth. Lung tumors were inspected at approximately 18 weeks post-inhalation. Pieces of lung tumors were taken from transgenic mice and were implanted subcutaneously (without any in vitro manipulation) into Scid/beige mice using trocar to generate KPT (Kras^G12D-LSL^p53^fl/fl ^trocar) model as described in the Materials and Methods. **B **Tumor implantation results in increased levels of OPN in the plasma in tumor bearing mice. **C **Using flowcytometry, expression of CD44v6 and αvβ3 was evaluated in KP cells and mPBMCs. Cells were stained with the antibodies as described in materials and methods and data analysis showed greater expression of αvβ3 than CD44 in both KP and mPBMCs. **D **KPT mice were randomized and received treatments (Vehicle, AOM1, Carboplatin and combination) at 8 days post-implantation. Tumors volume were measured twice/week and study was terminated at 27 days after implantation.

The Kras^G12D-LSL^p53^fl/fl ^GEMM (genetically engineered mouse model) represents one of the most relevant models of human NSCLC [41]. Biology of tumor progression and efficacy of therapeutic agents have been extensively studied in this model. Intranasal inhalation of viral particles containing Cre-recombinase results in activation of mutated KrasP^G12DP ^and ablation of p53 that in turn lead to tumor formation and progression in the lung reminiscent of lesions observed in cancer patients with a similar mutation [42]. Therefore, the availability of these mice prompted us to test efficacy of AOM1 on tumor growth and progression. However, repeat-dose treatment of these immuno-competent mice with AOM1, a fully human IgG2, resulted in rapid clearance of the antibody from plasma possibly due to the development of anti-drug antibodies (no changes in AOM1 clearance was observed following repeated treatment of immune-compromised mice, data not shown). To circumvent this limitation, we modified this tumor model by *de novo *isolating tumors from the lung of Kras^G12D-LSL^p53^fl/fl ^GEMMs and implanting them subcutaneously (without any *in vitro *manipulation) in immunodeficient scid mice to create KPT (Kras^G12D-LSL^p53^fl/fl ^Trocar) mice. All the implanted tumors were capable of growth and proliferation in the immunodeficient recipients (Figure 4A). ELISA data showed elevated levels of OPN in plasma in KPT mice suggesting a role for OPN in tumor progression in this model (Figure 4B). FACS data indicated that both tumor cells and PBMCs isolated from animals bearing these tumors express αvβ3 and CD44 receptors further supporting a rationale for treatment of sc-tumors with AOM1 (Figure 4C). Analysis of sc tumor volumes did not reveal any significant difference at the primary site of tumor growth in any of the treatment groups (including AOM1 as single agent or in combination with Carboplatin) suggesting that OPN may not play an important role in tumor growth at the primary site of tumorigenesis (Figure 4D).

### Lung metastasis is induced by OPN in KPT mice

**Figure 5:**
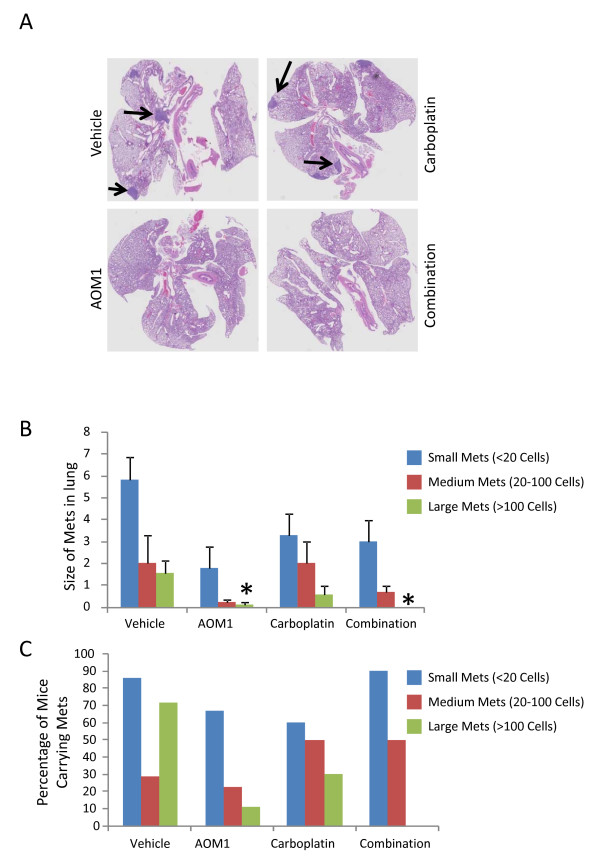
**AOM1 inhibits growth of large tumors in the lung in a NSCLC tumor**. **A **Scid/beige mice were sc implanted with pieces of tumors isolated from lung lesions from Kras^G12D-LSL^p53^fl/fl ^mice. Implanted mice were randomized at 8 days post-implantation and were treated with vehicle, AOM1, carboplatin and combination of both compounds. Tumor volume was measured using caliper twice per week. At terminal analysis whole lung from each mouse was fixed in formalin and was stained in H&E. Representative images from each treatment are shown. In pathology analysis lung lesions were classified into small (less than 10 cells) medium (10-200) and large (more than 200 cells) size and were quantified in each treatment. **B **Quantifications of lesions in each treatment. Bar graph represents mean number of lesions ± SEM. **C **Frequency of mice carrying each lesion in each treatment also indicated that AOM1 as single agent or in combination with Carboplatin significantly inhibits percentage of mice carrying large tumors in the lung.

In addition to primary tumor growth, the sc-implanted tumors had the capacity to metastasize to the lung indicating that tumor pieces from the GEMMs have maintained their invasive capacity. We analyzed metastasis in the lungs and further classified tumor lesions as small, medium, and large according to the size of the lesions (Figure 5A). Pathology analysis indicated that while there was no significant difference in the number of small or medium tumors in the lung, AOM1 as single agent or in combination with Carboplatin significantly inhibited growth of large tumors (Figure 5B). In addition analysis of the frequency of lung metastases showed a significant decrease in the percentage of mice carrying large lung tumors following treatment with AOM1 as compared to the vehicle-treated animals, particularly in combination treatment group (AOM1 plus Carboplatin) where none of the mice carried large tumors as judged by the histological analysis (Figure 5C). These observations suggest a role for OPN as a mediator of metastasis in a preclinical model of NSCLC.

## Discussion

Among molecular mediators of tumor growth and progression, OPN represents a complex target/pathway particularly in drug development. OPN has been identified in several pathological tissues (inflammatory, obese, and cancerous) in the organism [1]. OPN expression is elevated during inflammation to recruit macrophages and other immune infiltrating cells. A recent report shows that OPN may play a significant role in obesity through regulation of insulin signaling in liver cells and inflammation [43]. In cancer, OPN is highly expressed in a variety of tumors and appears to be a prognostic factor correlating with tumor progression in patients. Despite wide expression and involvement in multiple pathological conditions, the lack of OPN in mice is not embryonically lethal nor does it causes a prominent phenotype compared to wild type mice suggesting that alternative mechanisms compensate for the lack of OPN or it may not play a key role in embryonic development [44]. One of the main challenges in characterizing role of OPN in tumor progression is the existence of two distinct families of receptors including integrins and CD44v6 that have the capacity to trigger downstream signaling pathways independent of each other. Therefore, inhibition of one of the two receptors/pathways may not completely suppress OPN signalling and development of therapeutic compounds to inhibit both receptors is extremely challenging if not impossible.

In the tumor mass, OPN is secreted by both stroma and cancer cells [36]. It appears that there are distinct functions for tumor-derived vs. stromal-derived OPN in tumor growth and metastasis. Crawford et al developed a model of cutaneous squamous cell carcinoma in OPN null mice and showed that while the number of metastatic tumors is increased in this model, the size of metastasized tumors was significantly lower compared to corresponding wild type mice [45]. It is suggested that stromal OPN may recruit anti-tumor macrophages resulting in smaller tumor growth [45]. However, other reports in melanoma [46] and breast [47] tumors suggest that host-derived OPN is important r for tumor growth and metastasis adding to the complexity of OPN in tumor biology.

Here, we developed an anti-OPN antibody capable of neutralizing human and mouse OPN, and utilized it to investigate the role of OPN in preclinical models with particular focus on lung cancer since a significant amount of data supports a role for OPN in NSCLCs [48]. All three transcripts of OPN have been identified in NSCLC patients and gain-of-function analyses indicate that OPNa, but not OPNb or OPNc, is involved in increased proliferation, migration, and invasion of tumor cells [49]. Serum OPN has been shown to act as a biomarker in lung carcinoma [38,50]. Conversely, reduction in serum OPN (e.g. due to resection of primary tumors) [51] is an indicator of better outcome in NSCLC patients treated with cytotoxic agent [52]. Despite all these reports, it remains to be clearly determined if OPN is a biomarker and/or a driver of tumor progression in NSCLC.

The Kras^G12D-LSL^p53^fl/fl ^mice [53] is one of the most relevant preclinical models of NSCLC since 20-30% of NSCLC patients carry Kras mutation [54] and 35-60% show genetic aberrations in p53 [55]. Capacity of tumor fragments to engraft in immuno-deficient animals provided an opportunity to test efficacy of AOM1 in NSCLC tumors. Lack of response to AOM1 in primary tumor growth indicates an overlapping mechanism between OPN and the other tumor-promoting factors. However, inhibition of the growth of metastatic lesions, which had been seeded prior to the initiation of AOM1 treatment, suggests a role for OPN as a mediator of metastasis rather than a regulator of primary tumor growth. Further investigation is needed to unravel details of the role of OPN in lung metastasis. For example, it remains to be determined if OPN promotes seeding of a specific clone of tumor cells that will eventually outgrow to large tumors in the lung or it is required to further promote tumor growth at late stage in the metastatic niche. Alternatively and given our *in vitro *data, OPN may inhibit migration and seeding of clone of tumor cells that may eventually rise to large tumors. Future work in this direction will likely result in an increased understanding of this complex protein that might have some benefits for cancer patients

## Abbreviations

OPN: Osteopontin; SPP1: secreted phosphoprotein; RGD: arginine-glycine-aspartate; AOM1: anti-OPN monoclonal antibody; NSCLC: Non-small cell lung adenocarcinoma; hPBMCs: Human peripheral blood mononuclear cells; ERM: ezrin/radixin/moesin; ECM: extracellular matrix; MMPs: matrix metalloproteinases; uPAs: urokinase plasminogen activator; VEGF: vascular endothelial growth factor; iNOS: inducible nitric oxide synthase; SPR: surface plasmon resonance; GEMM: genetically engineered mouse model; KPT: Kras^G12D-LSL^p53^fl/fl ^Trocar

## Competing interests

All authors are employees and shareholders of Pfizer.

## Authors' contributions

FS, NS, SB and EK designed experiments and contributed in execution of studies. XK, AF, SK, BS, AW, JL executed studies and PL provided pathology analyses. FS wrote the manuscript which was edited revised by FS, NS, AF, PL and EK.
